# The Independent Vitality of Blood, and the Comparative Merits of the Humoral Pathology

**Published:** 1855-12

**Authors:** L. G. Robinson


### BUFFALO MEDICAL JOURNAL

AND

### MONTHLY REVIEW.

**VOL. 11.** DECEMBER, 1855. **NO. 7.**

### ORIGINAL COMMUNICATIONS.

ART. I.—*The Independent Vitality of Blood, and the Comparative Merits of the Humoral Pathology.* (An Essay read before the Detroit Medical Society.) By L. G. Robinson, M. D.

In the second chapter of Genesis and seventh verse, we read as follows, viz: "And the Lord God formed man out of the dust of the ground, and breathed into his nostrils the breath of life, and man became a living soul."

All who give credence to these words of inspiration, find no difficulty in accounting for the origin of the human race. And taking it for granted that none of us are skeptical on this point, I find at once a premise or basis for reason, or deduction, worthy of anthropology in its application to scientific research. This graphic history of man's origin, mythical as it may appear to some, is strikingly verified in the analysis of his component parts—the ultimate elements of his integral body, as well as in those laws which resolve his symmetrical proportions into the dust of the earth. The successive steps of our Creator, in the work of man's formation, clearly indicate the two great divisions of his compound nature. The first step was the organic arrangement of those particles, called forth by the "fiat" of Omnipotence from the dust of the earth. This being accomplished, the framework and machinery of man was completed; and like every other work which is the work of

Omniscient design, it bore the stamp of perfection. Here, then, we first find man's physical nature—beautiful and perfect in its form, and in the adjustment of all its organs, but only a mass of inert matter, waiting the "fiat" of his Creator. The second step of this "crowning work of creation," was to breathe into his nostrils the breath of life, when "man became a living soul." He may here be compared (and not inaptly) to the "fœtus in utero," who, like the first man, is waiting for "the breath of life" to establish its independent vitality. The fœtus, however, is unlike the first man in this respect, that it has enjoyed for a time a state of vitality: not breathing itself, but being the recipient of the product of respiration in the arterialized blood derived from its maternal source. The phenomena presented by the fœtus receiving the breath of life, is familiar to every practitioner of midwifery. No sooner is the head born, than there is a rush of air down the air-passages, by which the cells of the lungs are suddenly and forcibly distended. From the violent crying of the child, it may reasonably be inferred that this act is a painful one, although the crying doubtless facilitates a first inspiration. Now who can say that the breath of life, as first given to man, differed, in any respect, from that given to his offspring? Hence, we see, that the communication of an atmosphere was all that was necessary for the evolution of vitality. And no reference is made, either directly or indirectly, in the sacred writings, to any other species of vitality than that derivable from the respiratory function alone.

To understand the respiratory processes upon the first man, we must suppose that the blood was *venous blood.* The action of the atmosphere upon it necessarily converted it into *arterial blood;* and as soon as this began to circulate (the lungs being the motive power) man awoke into being.

The venous blood in the capilliaries became converted into arterial, the blood began to move and passed into the pulmonary veins; the pulmonary veins conveyed it to the left side of the heart, and this organ, as soon as the quantity was sufficient, contracted upon its contents and established the systemic circulation. Thus blood was universally distributed throughout the whole body, exciting and stimulating the brain, and conveying life and warmth to every organ. Such must have been, so far as our philosophy extends, the vivifying processes of the first man. This statement may at first appear startling to those who are inclined to a more spiritual than physiological view of the subject. A little investigation of it, however, will fully justify this belief, for, if the blood were all arterial, the lungs at the moment of inflation, would have no function to perform, and it would be some time before they could have, inasmuch as the blood must at first circulate at least once through the body. If the arteries were filled with arterial, and the veins with venous, blood, that pre-supposes a circulation without the aid of the lungs as a consequence, therefore we are led to conclude that the veins were filled with venous blood, and the arteries were empty. We cannot but imagine, in truth, that the first man, prior to the inhalation of an atmosphere, in all respects similated death. The body was well and beautifully formed, but as in death, so in this man, the arteries were entirely empty, because in this instance they had never been filled, and circulation, therefore, would begin simultaneously with the first development of the respiratory function. The direct action of the air received into the lungs would be to oxygenate the blood, which by its agency would now be rendered vital, and, as a consequence, would begin to move. The heart would not be implicated in this first circulation of the blood, and there need be no difficulty in understanding this part of the supposition, because we know that the blood circulates in all animals, not excepting man, at some period of their being, prior to the formation of a heart. In this instance the circulation must have existed only between the lungs and the heart.

Now let us go back to the first development of a blood-vessel and its contents, and see what it is.

In the initial development of the ovum, we see a number of nucleated cells, arranged in a lineal series, and it is important here to observe, that these cells differ, in no respect, from the remainder of the cells of which the yolk is composed. These cells begin to absorb at their points of junction, so that two cells become formed into one, and by continuing this principle of operation a tube is gradually formed and indefinitely extended. It will be well to observe, also, in passing, the remarkable parallelism existing between the development of the tissues of the higher plants and animals. The lower plants consist of a more or less dense congeries of simple nucleated cells; but the higher plants require the development of *organs*, by means of which the several compound functions of the organism may be fitly carried out. These super-added organs are for the most part a system of tubes. Thus we have spiral vessels, which constitute the vascular system of plants and super-added milk vessels, &c. Now in all instances these tubes have resulted from a process precisely similar to that in which blood-vessels originate in the animal kingdom, viz., by the absorption of the points of contact, of a system of cells arranged in a lineal series, and a much *elongated cell*, or tube, is the result.

One of the examples in the animal kingdom, where the tissue retains its primitive type of organization through life, is worthy of special mention. This tissue is cartilage, which firstly formed on the principle of true development from cells, continues to be maintained throughout life: on the same principle, as fast as a portion of the cartilage is removed by the action of the absorbents it is renewed by the development of cells from exciting cells. But to return to the development of blood-vessels, we will inquire what becomes of the *nuclei* which the original cells contained?

They here become the first blood-corpuscles, and by the law of spontaneous division, which so eminently characterizes the nuclei of cells, continue to produce other blood-corpuscles; and the process goes on, in all probability, until the period of the first circulation of the whole fluid, which very speedily follows the above-described processes. Up to this point we clearly see that the blood is certainly *vital*, because it possesses the power of propagation. But it is only a *vegetable vitality.* There is no more vitality in any part of this fluid than in any other cells of which the vitelline mass is composed. In other words, it *is as vital*, but *no more vital*, than the rest of the structure; and I would here ask, if this condition of things changes, at any time, or under any circumstances?

The same remark applies with equal force to another most important organism, which certainly controls, to the greatest degree, the whole of the vital processes—*the nervous system.* If we derange the balance between the brain and the circulatory apparatus, that brain sustains a death, which is more or less temporary, as the circumstances may direct. The nervous system is at least as vital as the blood, and yet if the circulation be arrested, its vitality ceases. No one will be disposed to repeat the testimony which the *microscope* affords in its positive relations upon this question. Examine, then, microscopically, fresh drawn blood. Do we see any evidence of independent vitality? Immediately, 't is true, we see motion; but what causes it? simply the motion of the solid corpuscles in the liquor-sanguinis, produced by the agitation and disturbance of the whole, differing in no wise from what takes place with solid particles of matter suspended in water, and subject to the like agitation of the mass. In the same time both fluids will become equally quiet. It is obvious, then, that this motion alone can furnish no just claim to the appellation of a vital phenomenon. That either it is not an essential attribute of vitality, or can only exist as such, when associated with other attributes. In all its definite and descriptive analysis of blood, the microscope fails to furnish any better proof, or higher evidence of vitality. On the contrary, it shows that, removed from the influence of the nervous system, like the amputated limb, it dies instantly. The more we investigate this subject the more conclusive it will appear that there is no such thing as independent vitality in any organ. That vitality depends upon the due combination of all the vital organs; and if there be only the slightest separation of them, death is the immediate consequence. Amputate a portion of a nerve, as in operations of neurotomy, and it becomes a dead, and no longer a living nerve. Remove blood from the body, and it also is dead. I have already referred to the strictly vegetable character of human organization at a definite period of time. I have also shown how immediately animal structures die when separated from their connections. The question may now fairly be asked, does the same reasoning apply to vegetable tissues? To which we can but answer in the negative.

Vegetable tissues, unlike animal structures, consist entirely of congeries of cells, and especially in the lower orders of plants, each cell constitutes the plant, each cell under any circumstances maintains its own individual vitality, irrespective of the health of surrounding cells. This fact may be well illustrated by example: An apple falls from a tree, and one portion of it comes in violent contact with the ground. All those cells subject to concussion die, but the vitality of those cells which compose the remainder of the apple, remains unimpaired, and to consume it for food we are compelled to masticate, that the saliva may destroy the vitality of the cells, in order that the stomach may digest it; for it is a well-known fact that so long as vegetable cells or living animals retain their vitality, the stomach has no power for them: its function only begins when the vitality of the food ceases. And here we are met with another and important query. How is it that vegetables can maintain their living principle for so long a period of time, when an animal, or portion of an animal, dies instantly? The sole answer to this question will be found in the possession of a nervous system which belongs exclusively to the animal kingdom.

Such is the amount of controlling power, which even the most obtuse nervous system possesses, that all the vital processes are directly subject to its impulses. The more energetic the nervous system, associated with a high condition of the respiratory function, and a perfect aeration of the blood, as we find it developed in the warm blooded animals, the more readily is life destroyed. Whereas in the cold blooded reptiles, and many fishes, in which the nervous system and respiratory organs are but lowly organized, and the blood therefore impure, it is difficult to know when life is extinct.

If we remove the heart and chief blood-vessels from a frog, he can as well jump from the table — and with his stomach and intestines trailing on the floor, will continue hopping about with apparent unconcern. If we descend still lower in the scale of being, we can do as *Trembly* did, mince a polyp to atoms, and each fragment will become a perfect animal, forming another illustration of the vegetable character of animal life. I have mentioned these facts with a view to establish the great significance of the nervous system, and also to show that it is entitled to preëminence as compared with the blood. That vitality exists in direct ratio to the development of the nervous structure, and that the degree of vitality, inherent in the blood of any animal, is dependent upon the sensibility of the nervous system alone.

Now if these facts be admitted, let us see how far they can be applied to sustain the doctrines of the humoral pathology. The great argument of the humoralist, is the *independent vitality of the blood;* and to this he attaches the utmost importance. According to his theory, when a man is innoculated with any poison, the blood instantly becomes vitiated, as by some spiritual affinity, and with great activity transmits the obnoxious substance throughout the body. Now may we not find a more rational theory for this phenomena by making the absorbent system responsible solely for taking up and diffusing morbific matter? What are the absorbents, and what their relation to the circulation of the blood?

We hear enough about them in the discussion of medical questions to entitle them to more respect than we are apt to pay them. They are, as we know, universally distributed throughout the whole body, and to such an extent as to vie in number with the blood-vessels themselves. Dissections even by the microscope, fail to detect them. Their contents are diaphonous to the highest degree, and the injecting syringe has scarcely more power over them than the scalpel. A very minute and successful injection will display the vessels of the lymphatic glands. But the only mode of exhibiting the absorbent vessels at all is through the agency of mercurial injections. The absorbent system of a frog, as we learn, is remarkable for the possession of four distinct cavities, called lymphatic hearts, and these may be seen distinctly to pulsate through the external skin. How many such corresponding sacs there are connected with the absorbent system of man, we know not, still the fact is interesting and valuable as confirming the complete identity and isolation of this important system. While conveying a poison through the body the absorbents become most conspicuous, and appear to be in a state of inflammation. Their parieties are enlarged, and those which are most superficial, are readily seen through the skin, as a series of intensely red lines. In the case of a dissecting wound, for example, the absorbent system of the arm up to the axilla, is clearly demonstrable and beautifully displayed by the intense redness which characterizes it during the transmission of the poison. The veins, under the same circumstances, are not influenced by the poison, and of course those in immediate connection with the capillaries are small enough to display the effect if it existed, they being much smaller than the trunks of the absorbents themselves. There is no evidence, therefore, that the vascular system is at all implicated in the general effect, and if the blood be examined by the microscope, neither *does it* exhibit any more in this than any other diseased condition of the body, the slightest evidence thereof.

Again, the introduction of morbid matter into the blood must necessarily increase the specific gravity of the liquor-sanguinis, whereby the corpuscles of the blood would have great difficulty in circulating at all; and if the degree of absorption were sufficient, circulation would be altogether suspended. Whereas we know that under such circumstances the circulation is accelerated.

What takes place in fever? The nutritive function is arrested. The liquor-sanguinis, for want of its necessary pabulum, becomes thin and watery, and the flow of the solid particles is greatly augmented, which can only result as a consequence of disturbing the equilibrium of the liquor-sanguinis. Under healthy circumstances nature, as we know, provides against the contingency of increased specific gravity of the liquor-sanguinis by too great a supply of fibrin and albumen held in solution, by rolling them up into discs, and in this condition they constitute the colorless blood corpuscles. To this argument I attach much importance, showing the improbability, if not the impossibility, of the blood-vessels being the recipients of any contagious or other morbific materials. That the blood is not always and equally healthy, we admit. We know that in a languid and prostrate condition of the nutritive function, that the blood gradually deteriorates: that there is a want of fibrinous and albuminous elements; that the liquor-sanguinis is thin and watery; that the colored corpuscles are paler in color, and fewer in number.

But with regard to the consequence of poisons, at best the blood can only be remotely affected, for the process is clearly the following: the poison is taken up and extensively distributed by the absorbent vessels. These vessels ramify to all parts of the body, everywhere along their course are to be found the so-called lymphatic glands, and they, in the aggregate, amount to millions. Now who knows what is the function performed by these glands? Who can say what is the modifying or other influence which they exert? It can only with certainty be said, that hitherto their function has altogether eluded detection. They remain as a sealed book to the physiologist. They possess, however, one obvious and striking peculiarity, that while they transmit through their interior the vessels of the absorbent system, their external surface is covered with a most minute capillary plexus derived from the systemic vessels.

But while we know nothing of the specific action of lymphatic glands, which must be very peculiar, we do know the function of the absorbent system as a whole. To the absorbents is delegated the power of constantly renewing the body. Every tissue is so far subject to their influence that it is in all its parts gradually annihilated, the product being conveyed into the general circulation by the absorbent vessels, transmitted to the lungs and then thrown off and also discharged by exhalations from the skin secretions, &c. Simultaneously with the destruction of parts, is the deposition of new matter by the same organs—still we know nothing of the share which lymphatic glands take in these processes. We can only suppose that every form of matter submitted to them is subject to considerable elimination, and this fact we derive from their pathological rather than their healthful condition. In scrofula, for example, these glands are prone to extensive disease, and oppose themselves more to curative means than any other structures. But the best example of the capabilities of lymphatic glands is met with where a scrofulous patient contracts venereal disease. Once innoculated with the virus, they digest it, so to speak, and retain it, continually circulating through the connecting absorbents an evil influence, that almost, and often does, defy human skill. Commencing in the genital organs the poison is transmitted from one cluster of these glands to another, till remote parts are implicated in the mischief, and in such condition the natural function, as we may reasonably infer, is much interfered with. One of the most common effects of the venereal virus, is a removal of *bones*, or parts of bones. Now upon what physiological action is this to be accounted for?

As I believe only upon the following: That while under any circumstances the absorbent system retains its power to remove parts, it has lost the capacity of renewing them by the deposition of healthy material. In other words, the lymphatic glands being themselves diseased through the venereal poison, necessarily confer a taint upon every thing with which they come in contact, and as old bones cannot be repaired with morbid material they must suffer loss. Also in diseased mesenteric glands they must necessarily wholly stop the transit of nutrient material. It matters not how nutritious the diet, the patient must die by a slow lingering death, for these glands as much interfere with the flow of nourishment, as in the explanation already given of the destruction of bones in venereal diseases.

But to return to our illustration of the poison imbibed from a dissecting wound merely to show what becomes of it. It appears that the poison is conveyed by the absorbents to the *thoracic duct*, and then for the first time it comes in contact with the general circulation: but whether the grosser particles of morbific matter, or some equally fatal, though etherial emanation of it, we know not, we can scarcely doubt, however, that the poison is subject to a process of elaboration in its transit through the lymphatic glands. The chemical principle is undoubtedly there, but its presence *is amorphous,* so that as already observed, even the microscope fails to detect it.

In what manner the mind of man is connected with the body, "doth not yet appear;" but that a great part of the nervous system is subservient to and under the control of the mind, is clearly demonstrable. Indeed if we could believe that any one part of the body was made expressly, and exclusively, for the mind, we should say it was the nervous system, the subtilty of its laws seem so nearly to similate the silent workings of the mental faculties. The mind furnishes to disease many avenues, and invites it to exercise its laws and carry on its destructive operations in the body through the agency of the nervous system.

For example: anger, which all will admit is a mental phenomenon, communicates primarily with the nervous structure, through *it* increases the circulation even to a degree of inflammatory action. Grief acting upon the nervous energies prostrates the action of the vessels and undermines the whole body. In short, the exercise of all the attributes of the mind, when excessive, may so disturb the equilibrium existing between the mind and the body, as to be partly regarded as the primary exciting cause of diseased action.

If, then, all diseases have their origin in the blood and other fluids of the body, the mental emotion must be of humoral origin, a conclusion as absurd as the premises from which it is drawn. The light which the science of physiology clearly throws upon this question, leads us to the conclusion that when our Creator first gave to man "the breath of life," He gave vitality not to the blood alone, neither made *it alone* the dispenser of life, no more than to the nervous system and other tissues of the body, but He gave it to the body-corporate, the harmonious union of all in one.

